# Optical coherence tomography-based assessment of retinal vascular pathology in cerebral small vessel disease

**DOI:** 10.1186/s42466-020-00062-4

**Published:** 2020-05-15

**Authors:** A. Abdelhak, A. Huss, A. Brück, U. Sebert, B. Mayer, H. P. Müller, H. Tumani, M. Otto, D. Yilmazer-Hanke, A. C. Ludolph, J. Kassubek, E. Pinkhardt, H. Neugebauer

**Affiliations:** 1 Department of Neurology & Stroke, University Hospital of Tübingen, Tübingen, Germany; 2 Department of Neurology, University Hospital of Ulm, Ulm, Germany; 3 Institute of Epidemiology and Medical Biometry, Ulm, Germany; 4 Specialty Clinic of Neurology Dietenbronn, Schwendi, Germany; 5 Clinical Neuroanatomy Section, Department of Neurology, Ulm, Germany; 6 Department of Neurology, University of Wuerzburg, Würzburg, Germany

**Keywords:** Microangiopathy, OCT, Retinal vessels, Biomarker, SIMOA, Small vessel disease

## Abstract

**Background:**

Cerebral small vessel disease (CSVD) is a disorder of brain vasculature that causes various structural changes in the brain parenchyma, and is associated with various clinical symptoms such as cognitive impairment and gait disorders. Structural changes of brain arterioles cannot be visualized with routine imaging techniques in vivo. However, optical coherence tomography (OCT) is thought to be a “window to the brain”. Thus, retinal vessel parameters may correlate with CSVD characteristic brain lesions and cerebrospinal fluid biomarkers (CSF) of the neuropathological processes in CSVD like endothelial damage, microglial activation and neuroaxonal damage.

**Methods:**

We applied OCT-based assessment of retinal vessels, magnetic resonance imaging (MRI), and CSF biomarker analysis in a monocentric prospective cohort of 24 patients with sporadic CSVD related stroke and cognitive impairment. MRI lesions were defined according to the STandards for ReportIng Vascular changes on nEuroimaging (STRIVE). Biomarkers were assessed using commercially available ELISA kits. Owing to the unavailability of an age-matched control-group lacking MRI-characteristics of CSVD, we compared the retinal vessel parameters in CSVD patients (73.8 ± 8.5 years) with a younger group of healthy controls (51.0 ± 16.0 years) by using an age- and sex-adjusted multiple linear regression analysis model.

**Results:**

Among the parameters measured with OCT, the Wall to Lumen Ratio (WLR) but not Mean Wall Thickness (MWT) of the superior branch of the retinal artery correlated significantly with the volume of white matter hyperintensities on MRI (r_s_ = − 0.5) and with CSF-levels of Chitinase 3 like 1 protein (r_s_ = − 0.6), zona occludens 1 protein (r_s_ = − 0.5) and GFAP (r_s_ = − 0.4). MWT and WLR were higher in CSVD than in controls (28.9 μm vs. 23.9 μm, *p* = 0.001 and 0.32 vs. 0.25, p = 0.001).

**Conclusions:**

In this exploratory study, WLR correlated with the volume of white matter hyperintensities, and markers of vascular integrity, microglial activation, and neuroaxonal damage in CSVD. Further prospective studies should clarify whether retinal vessel parameters and CSF biomarkers may serve to monitor the natural course and treatment effects in clinical studies on CSVD.

## Introduction

Cerebral small vessel disease (CSVD) is a common cause of stroke and dementia in elderly individuals with poorly managed cardiovascular risk factors [28]. While magnetic resonance imaging (MRI) can capture the end results, such as white matter hyperintensities (WMH), the underlying pathophysiological mechanisms, and especially changes in small cerebral vessels, remain unexplored in in vivo studies [42, 43]. The pathophysiology of CSVD involves vessel wall thickening and bagging, hypoxic damage, breakdown of the blood-brain barrier, microglial activation, astrogliosis, and neuroaxonal demise [14, 22]. Similarities between the retinal vessels and small cerebral vessels are numerous and have been described extensively [24]. Therefore, the assessment of retinal vessels may offer a unique opportunity for in vivo analyses of vessel pathology in CSVD. The association between retinal microvascular changes and CSVD was reported before [16, 19, 45]. However, the employed retinal fundus imaging analysis may not have assessed all aspects of retinal vessel changes: the previous studies mainly assessed total vessel diameters and presented the arteriole to venule ratio, but did not report the thickness of the wall or the lumen diameter of the retinal arterioles [15, 44]. Similar limitations apply to the assessment of retinal vessels using flow-dependent scanning laser Doppler flowmetry [4, 32].

In our study, we used a novel non-invasive optical coherence tomography (OCT) technique to visualize and measure the diameters of the retinal vessel from the routine ring scans [30] in patients with symptomatic sporadic CSVD. To the best of our knowledge, the reports applying this method to assess the retinal vessels are scarce. Besides the original paper reporting the results in healthy individuals [30], only one study assessed the retinal vessels in patients with amyotrophic lateral sclerosis using the same method [2]. Our central hypothesis was that changes in anatomical parameters of retinal vessels will correlate with the increase in the load of characteristic brain lesions of sporadic CSVD on MRI and with changes in cerebrospinal fluid (CSF)-biomarkers of the underlying pathophysiological processes of disturbed vascular integrity, astrogliosis, microglial activation and neuroaxonal demise (Supplementary Figure 1). Hereby, we focused on 1) zonula occludens-1 (ZO-1), a novel marker of blood-brain barrier disruption that is often associated with glial pathology in CSVD (A [8, 29].), 2) glial fibrillary acidic protein (GFAP) as a marker of astrogliosis [37], 3) phosphorylated neurofilament heavy chain (pNfH) as a marker of neuroaxonal damage [36], and 4) chitinase-3-like-1 protein (CHI3L1, alias YKL40) that reflects microglial/ macrophage activation [3, 5, 21]. At this time, little is known about the levels of these CSF-biomarkers in CSVD: elevated CSF concentrations of GFAP were found in other cerebrovascular diseases like intracerebral bleeding and ischemic stroke [18]; only serum concentrations of CHI3L1 were reported previously in acute ischemic stroke [9, 23]; and higher levels of pNfH were reported in various neurological disease, including in the serum of stroke patients [36].

## Methods

### Patients selection

Patients with sporadic CSVD related stroke and vascular cognitive impairment were prospectively recruited for the study at the stroke unit and the memory outpatient clinics of the University Hospital of Ulm, Germany. Sporadic CSVD related stroke was diagnosed after exclusion of other stroke etiologies by thorough cerebrovascular workup including duplex-sonography or computed tomography (CT)-angiography of brain supplying arteries, transthoracic echocardiography, and transesophageal echocardiography if indicated, continuous electrocardiography (ECG)-monitoring for at least 72 h and additional holter ECG in high-risk patients for atrial fibrillation (i.e., enlarged left atrium, high rate of supraventricular extrasystoles during continuous monitoring), routine blood tests and past medical history negative or not suggestive for hereditary cerebrovascular diseases. Patients discharged from our stroke unit were followed up months later in our outpatient clinic. Degenerative dementias, in particular Alzheimer’s Disease (AD), as well as other causes of cognitive impairment such as metabolic and endocrinological disorders, were ruled out by neuropsychological examination, extended laboratory investigations, and lumbar puncture. Each patient’s history was reviewed, in particular concerning the previous antihypertensive treatment and pre-existing retinal disease, blood pressure was measured, and OCT scans were finally performed. Only patients with complete datasets, including OCT scans of both eyes, were included in the analysis. Patients with a history of conditions that may affect the structure of the retinal vasculature beyond the risk factors of CSVD were excluded.

To show that retinal vessels in CSVD are altered compared to a situation before the onset of CSVD, we used group-level data from 20 healthy adults that were obtained using the same OCT-method and that were published before by our group [2].

### OCT examination and assessment of the retinal arterioles

OCT examination was performed using spectral domain (SD)-OCT (Spectralis platform, Heidelberg Engineering, Heidelberg, Germany: software version 6.2). Retinal vessels were visualized through a 3.4-mm circular scan around the optic nerve, with a minimum of 50 automatic real-time measurements, according to OSCAR-IB criteria [40]. We analyzed the superior temporal branch of the retinal artery at its crossing on the 12° circular scan using volume of interest-based analysis, according to the method described by Rim et al. [30]. In the study, we included the mean values of inner or outer wall thickness from both eyes (IWT and OWT, respectively). The mean wall thickness was calculated from the mean values of the IWT and OWT. The wall-to-lumen ratio (WLR) was calculated by dividing the mean wall thickness of the superior temporal branch of the retinal artery by the lumen diameter (LD); this was applied as a composite measure of the vessel pathology [4, 10, 20, 30, 32].

### Lumbar puncture

Lumbar puncture (LP) was performed for diagnostic purposes only with written informed consent from all patients. CSF and serum samples were collected on the same day and stored according to the consensus protocol for the standardization of CSF collection and biobanking [39]. Blood contaminated CSF samples were excluded.

### CSF-biomarker profile

The microglial activation marker chitinase-3-like protein1 (CHI3L1) levels were measured using a Human CHI3L1 Quantikine enzyme-linked immunosorbent assay (ELISA) kit (R&D Systems, Inc., MN, USA). For astrogliosis, glial fibrillary acidic protein (GFAP) was measured using the single molecule array (SIMOA) technology (Quanterix Corporation, Lexington, MA, USA). As a marker of axonal demise, phosphorylated neurofilament heavy chain (pNfH) were quantified with a commercially available ELISA (Biovendor, Heidelberg, Germany.) As a marker for endothelial damage, Zonula occludens protein-1 (ZO-1) levels were quantified using the Human tight junction protein-1 ELISA Kit (CUSABIO TECHNOLOGY LLC, Wuhan, China). All measurements were performed according to the instructions provided by the manufacturers.

### MRI scans

MRI data were acquired using a 1.5 T scanner (Symphony, Siemens Medical, Erlangen, Germany) to quantify WMH. Altogether 49 T2-weighted coronal slices (Fluid Attenuated Inversion Recovery/FLAIR, TR/TE 8500/82 ms) of 3.0 mm thickness, 0.43 × 0.43 mm2 in-plane resolution, and 512 × 448 voxels matrix dimension were scanned. Neuroimaging findings of CSVD were defined according to the STandards for ReportIng Vascular changes on nEuroimaging (STRIVE) [42, 43].

**Fig. 1:**
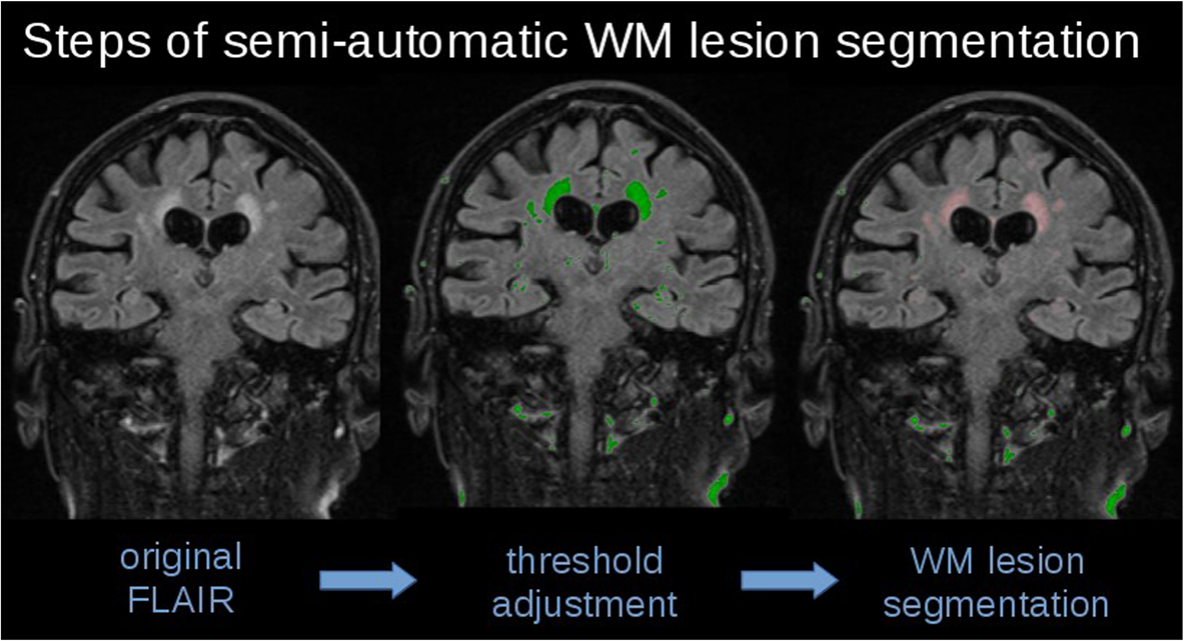
Steps of semi-automatic white matter (WM) lesion segmentation in one of our patients (central coronal slice): Lesions in the original fluid-attenuated inversion recovery (FLAIR) image appear as hyperintensities. After threshold adjustment (green voxels), WM lesions could be segmented (light red voxels)

**Fig. 2:**
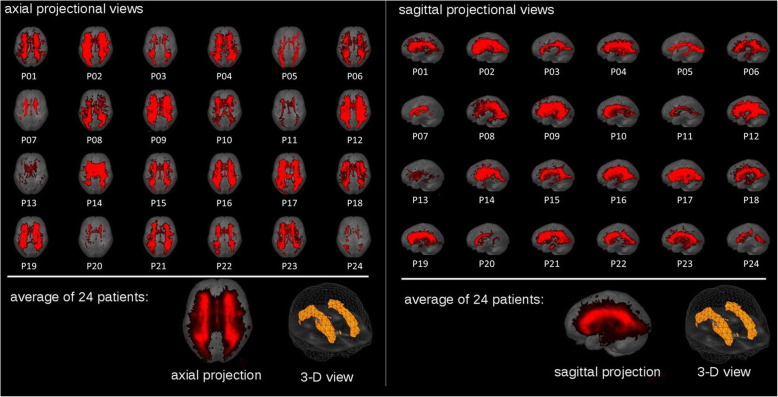
White matter hyperintensities (WMH) for 24 patients detected from FLAIR-MRI-recordings in axial (left) and sagittal (right) projectional views. For comparability, individual results were normalized to the Montreal Neurological Institute (MNI) coordinate frame and displayed on the same morphological background (segmented averaged brain)

The T2-weighted images were analyzed by an in-house software based on a semi-automatic image intensity analysis to calculate the volume of white matter hyperintensities (WMH) [27, 35]. The threshold for selecting WMH voxels had to be adjusted operator-dependently as T2-weighted MR images intrinsically represent no absolute intensity values (Fig. 1). In order to identify lesion-related voxels, voxels within an operator-defined intensity range were identified slicewise. The number of detected voxels (total amount of voxels corresponds to the total volume of the lesion) was identified as the WMH. WMH for the 24 individuals is summarized in Fig. 2 as axial and sagittal projectional views. Based on STRIVE definitions [42, 43], we defined a cerebral microbleed as a focal area (< 10 mm) of low signal intensity on gradient echo. Lacunes were defined and counted as ovoid or round, fluid-filled CSF-cavities in the subcortical areas, with a diameter between 3 and 15 mm, and surrounded by a hyperintense rim on FLAIR sequences. Visible Virchow-Robin perivascular spaces were identified as linear and round or ovoid spaces following the course of a vessel with a perpendicular diameter < 3 mm and without a hyperintense rim on FLAIR imaging.

### Statistics

All statistical tests were performed using SPSS® Statistics version 25 (IBM Corporation, Armonk, NY, USA) and R-Studio version 1.1 (www.r-project.org). All parameters were described descriptively employing frequencies or mean ± standard deviation (SD) or median (25% percentile – 75% percentile). The Shapiro-Wilk test, as well as graphical assessment by means of quantile-comparison-(QQ)-plots, were used to examine the distribution of continuous data. For skewed parameters, a log- transformation was performed. Student’s t-test was used to compare the mean values in the two groups of CSVD patients and young healthy controls (HCs). Where appropriate, one-way ANOVA was used to compare the mean values of more than two groups. In order to account for the small sample size and questionable normal assumption for some parameters, Spearman rank correlation was used to measure correlation.

Moreover, the results of the statistical tests mentioned above were verified using a multiple linear regression approach for each vessel parameter separately. Specifically, these models were applied to account for a possible confounding bias caused by imbalances between CSVD patients and HCs regarding age and sex. A *p*-value < 0.05 was considered statistically significant. Correction for multiple testing was not applied because of the exploratory character of our study. Figures were prepared using GraphPad Prism 7.04 software (GraphPad Software Inc., La Jolla, CA, USA).

## Results

### Clinical features

**Fig. 3:**
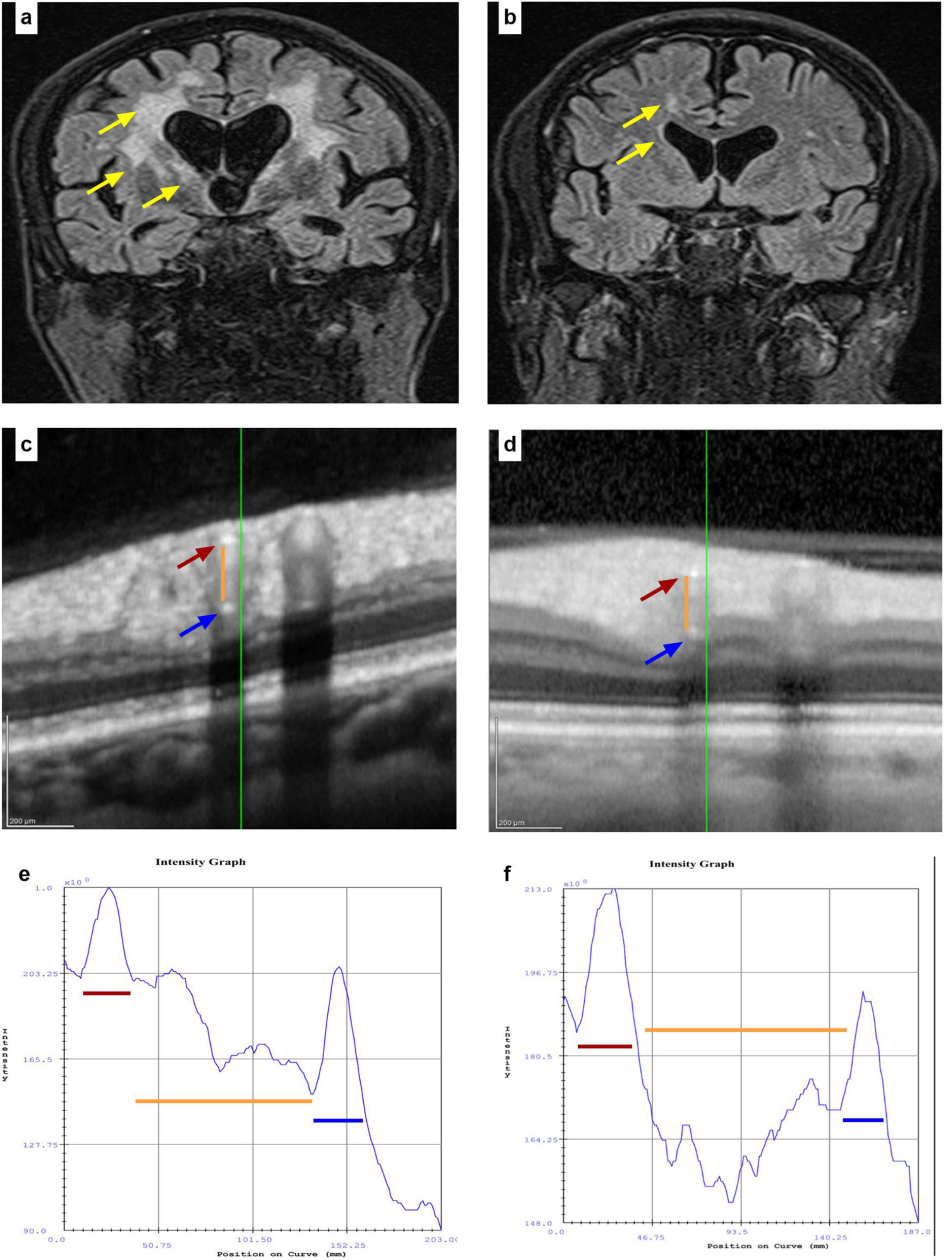
White matter hyperintensities (WMH) on FLAIR imaging and corresponding optical coherence tomography (OCT) scans and intensity graphs. **a** and **b** Examples of two patients with different extent of WMH on FLAIR imaging (yellow arrows), left Fazekas grade 3, right Fazekas grade 1, respectively. **c** and **d** OCT scans showing superior temporal branch of the retinal artery (red arrow: inner wall. Blue arrow: outer wall. Orange line: lumen). **e** and **f** intensity graphs showing the inner wall’s (red line) and the outer wall’s peak (blue line) with space in between (orange line) corresponding to the lumen

Between 2015 and 2017, we included 104 patients with sporadic CSVD related stroke and cognitive impairment that presented to our outpatient clinic. After exclusion of other causes for dementia and verification of persistent vascular cognitive impairment, 46 patients underwent retinal imaging with OCT. One patient was excluded due to eye disease (age-dependent macular degeneration) and one patient due to Cerebral Autosomal Dominant Arteriopathy with Subcortical Infarcts and Leukoencephalopathy (CADASIL). Twenty patients were excluded due to incomplete datasets. Finally, datasets from 24 patients with CSVD were included in our study. The clinical characteristics of the included patients are reported in Table 1. An example of the WMH with the corresponding OCT images is provided in Fig. 3.

**Table 1:** Summary of the clinical characteristics and concentrations of CSF-biomarkers assessed in *n* = 24 CSVD patients (continuous data given as mean ± SD unless otherwise stated)

Age (years)	73.8 ± 8.5
Sex	17 males, 7 females
Systolic blood pressure (mmHg)	157.9 ± 26.9
Diastolic blood pressure (mmHg)	84.5 ± 14.2
Low density lipoprotein (mmol/L)	2.3 ± 0.9
Glycated hemoglobin (HbA1c; mmol/mol)	39.4 ± 4.6
Number of antihypertensive medications	
One (n)	13
Two (n)	5
Three (n)	6
Mini mental state examination score (MMSE) (median, 25–75 percentile)	27 (24–28)
Magnetic resonance imaging lesion load (WMH) (mm^3^) (median, 25–75 percentile)	33.2 (13.5–45.9)
Number of Lacunes (median, 25–75 percentile)	2 (0.75–3.25)
Number of microbleeds (median, 25–75 percentile)	2 (0–7.0)
Visible Virchow-Robin perivascular spaces	
none (n)	5
present (n)	19
Fazekas-Score [13]	
One (n)	6
Two (n)	10
Three (n)	8
Chitinase 3 like 1 (CHI3L1) (ng/ml)	169.6 (± 60.4)
Glial fibrillary acidic proteins (GFAP) (ng/ml) (median, 25–75 percentile)	14.2 (10.1–18.4)
Phosphorylated neurofilament heavy chain (pNfH) (pg/ml) (median, 25–75 percentile)	663 (480–1022)
Zona occludens 1 (ZO-1) (pg/ml) (median, 25–75 percentile)	1005 (783–1377)

### Retinal vessel parameters in CSVD

Vessel parameters assessed in our CSVD patients are shown in Table 2. The majority of the retinal parameters studied showed significant differences between the CSVD cases and the young HCs (Table 2). In addition, IWT, MWT and total vessel diameter differed between males and females (*p* = 0.002, *p* = 0.018 and *p* = 0.001, respectively). There were also differences between antihypertensive treatment groups (receiving one, two or three medications) regarding mean lumen diameter (94.0 μM, 79.6 μM and 95.4 μM respectively, p = 0.002), and subsequently mean WLR (0.30, 0.37, 0,30 respectively, p = 0.018). The reported retinal parameters did not correlate with age.

**Table 2:** Comparison between patients with cerebral small vessel disease and healthy controls

Retinal vessel parameters (mean ± SD)	Cerebral small vessel disease n = 24	Young healthy controls *n* = 20	p-values (Welch two-sample t-test)
Inner retinal wall thickness (IWT) (μm)	30.1 (±4.5)	26.13 (± 4.7)	**0.002**
Outer retinal wall thickness (OWT) (μm)	27.8 (±4.0)	22.6 (±5.3)	**0.001**
Mean arterial wall thickness (MWT) (μm)	28.9 (±3.1)	23.9 (± 2.7)	**0.001**
Lumen diameter (LD) (μm)	91.3 (±9.2)	95.4 (± 9.6)	0.156
Wall to lumen ratio (WLR)	0.32 (±0.05)	0.25 (± 0.04)	**0.001**
Total vessel diameter	149.2 (±11.5)	143.2 (±11.9)	0.099

### Difference between CSVD patients and healthy controls

The young healthy controls differ significantly regarding age from our CSVD patients (mean age 51.0 (±16.0) years vs. 73.8 (±8.5) years (*p* < 0.001)) and sex distribution (15 females and 5 males in HCs vs. 7 females and 17 males in CSVD (*p* = 0.006). Following a simple and unadjusted comparison of CSVD patients and HCs, IWT, OWT, MWT, and WLR were significantly increased in CSVD patients, whereas a difference regarding lumen diameter and total vessel diameter could not be demonstrated (Table 2). In both groups, the interindividual MWT (right vs left) correlated positively (r = 0.5 in the CSVD patients and 0.4 in the HC, *p* < 0.01 and *p* = 0.04 respectively). No correlation was found for the lumen diameter.

The validity of the results reported in Table 2 was additionally assessed through a multiple linear regression model, including the group status (CSVD vs. HCs) as well as the potential confounders age and sex, which was run separately for each retinal vessel parameter. After adjusting for these confounders, MWT and WTR remained significantly different between CSVD patients and HCs (adjusted *p*-values were 0.008 and 0.006, respectively), which means that the changes in retinal vessel observed significantly depended on CSVD but not on age and sex.

### Correlative analyses in CSVD patients

**Fig. 4:**
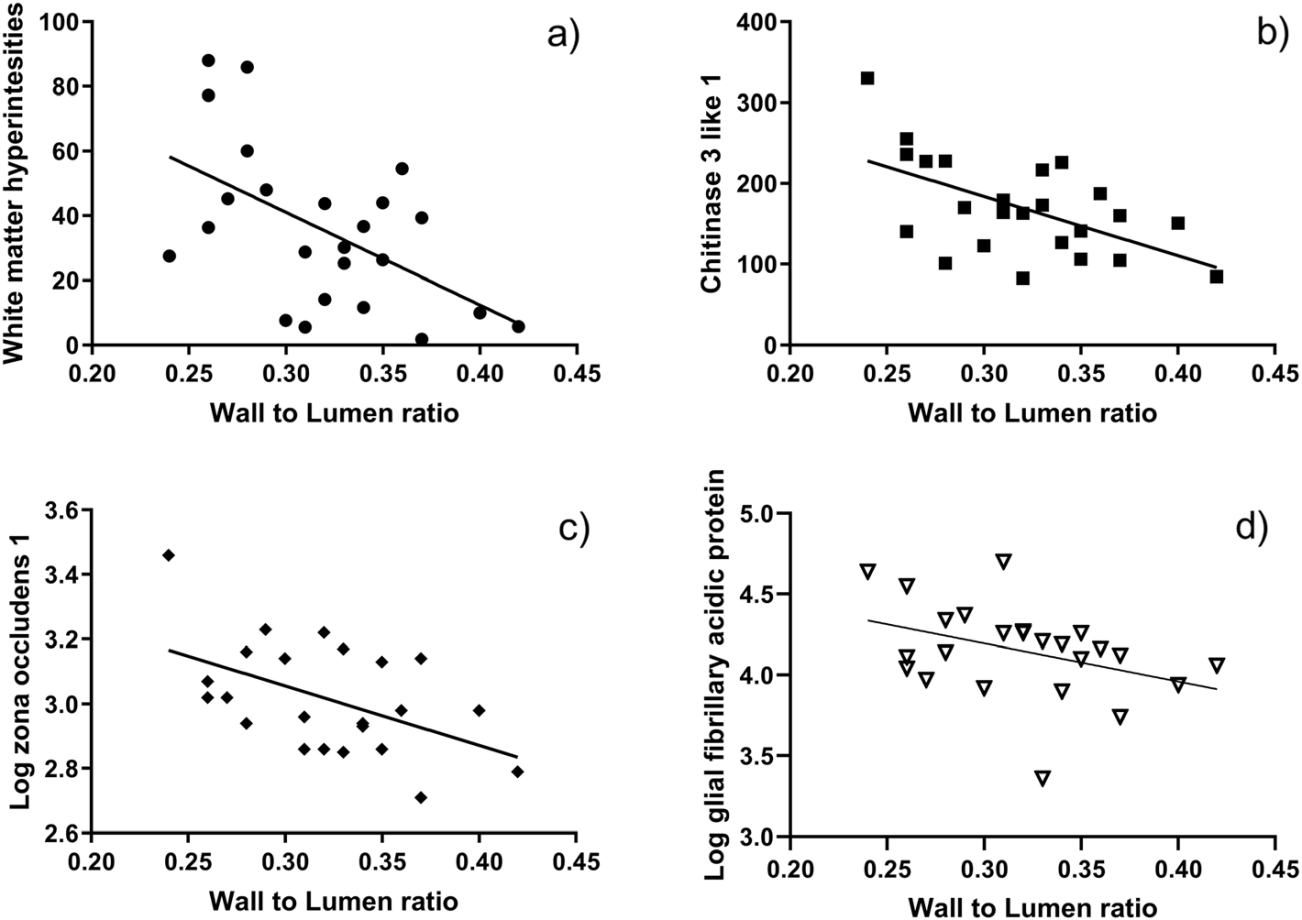
Correlations between wall to lumen ratio and white matter hyperintensities (**a**), CSF concentration of chitinase 3 like 1 (**b**), zona occludens (**c**), and glial fibrillary acidic protein (**d**) in CSVD patients

There was a moderate inverse correlation between WLR and the volume of WMH (r_s_ = − 0.5, *p* = 0.009, Fig. 4a). No further relevant correlation could be found between retinal vessel parameters and the included MRI parameters of CSVD.

Concerning retinal vessels parameters and CSF-biomarker (Fig. 4b,c), we found a moderate to strong inverse correlation between WLR and levels of CHI3L1 (r_s_ = − 0.6, *p* = 0.003), a moderate inverse correlation between WLR and ZO-1 (r_s_ = − 0.5, *p* = 0.02), and a weak to moderate inverse correlation between WLR and GFAP (r_s_ = − 0.4, *p* = 0.04). A similar weak to moderate inverse correlation was found between MWT and GFAP (r_s_ = − 0.4, *p* = 0.03, Fig. 4d).

Further correlation analyses using Spearman’s rank coefficient between retinal vessel parameters and both MRT and CSF-biomarkers are reported in Supplementary Table 1.

## Discussion

In this study, changes in retinal vessels were assessed using OCT in conjunction with characteristic MRI measurements of CSVD as well as markers of the disease, which are indicative of altered vascular integrity, astrogliosis, microglial activation, and neuroaxonal demise. We postulated a similar detrimental effect of common cerebrovascular risk factors such as arterial hypertension on the two embryonically related and structurally similar cerebral and retinal arteriolar vascular beds. As the intensity-based assessment of retinal vessels might underestimate the thickness of the outer wall, we focused on the WLR and MWT as a composite parameter reflecting the vessel pathology more accurately than the individual wall thicknesses [30]. The strong correlation between WLR and the volume of WMH on MRI in our study might support the hypothesis that retinal vascular abnormalities could serve as an indicator of the diagnosis and progression of CSVD. Increased WLR due to increased wall thickness and reduced luminal diameter of the retinal arterioles has been reported before in middle-aged patients with hypertension, transient ischemic attacks (TIA), and lacunar infarction, and has been described as a predictor of stroke and myocardial infarction [11, 31]. In accordance, our findings in old CSVD patients demonstrated an increased WLR compared to healthy young adults due to an increase in MWT.

In contrast to our expectations, we then found a consistently lower WLR in CSVD patients with a higher volume of WMH. A possible explanation is vessel dolichoectasia, which has been reported before in CSVD [26]. Dolichoectasia of intracranial arteries is characterized by thinning of the vessel wall and a simultaneous increase in the vessel lumen, which corresponds to a decrease in the composite parameter of WLR comparable to the findings in retinal vessels in our cohort. Intracranial arterial dolichoectasia is correlated with the imaging and histopathological evidence of CSVD [25, 26]. Another study reported an association between lumen dilatation of the middle cerebral artery and WMH [46]. The occurrence of intracranial arterial dolichoectasia may be explained by 1) loss of vascular endothelial integrity with fibrin deposition in the vessel walls resulting in increased arteriolar wall thickness ([34, 42, 43] and 2) degradation of structural proteins of the vessel wall by macrophages and microglia [33]. In coherence with this hypothesis, we found a strong correlation between WLR and ZO-1 as a marker of vascular integrity in CSF [17, 41].

The correlation with other CSF-biomarkers found here might reflect some of the histopathological changes in CSVD, which are mainly triggered by the vessel pathology [1, 22, 42, 43]. In particular, we found a robust correlation between retinal vascular changes and CSF-biomarkers that indicate glial activation (CHI3L1 and GFAP). Microglial activation was found in the periventricular and deep cortical white matter lesions in subjects with CSVD and might be induced by cerebral hypoperfusion [6, 12, 14, 47]. However, CSF levels of CHI3L1 should be interpreted with caution as CHI3L1 is not specific to microglial activation and maybe also released by activated microglia or macrophages/monocytes that enter the brain in response to damage as well as by reactive astrocytes [7]. Thus, more specific microglial markers such as sTREM2 [38] may be promising additional candidate markers for understanding the role of microglia in CSVD in future studies.

**Fig. 5:**
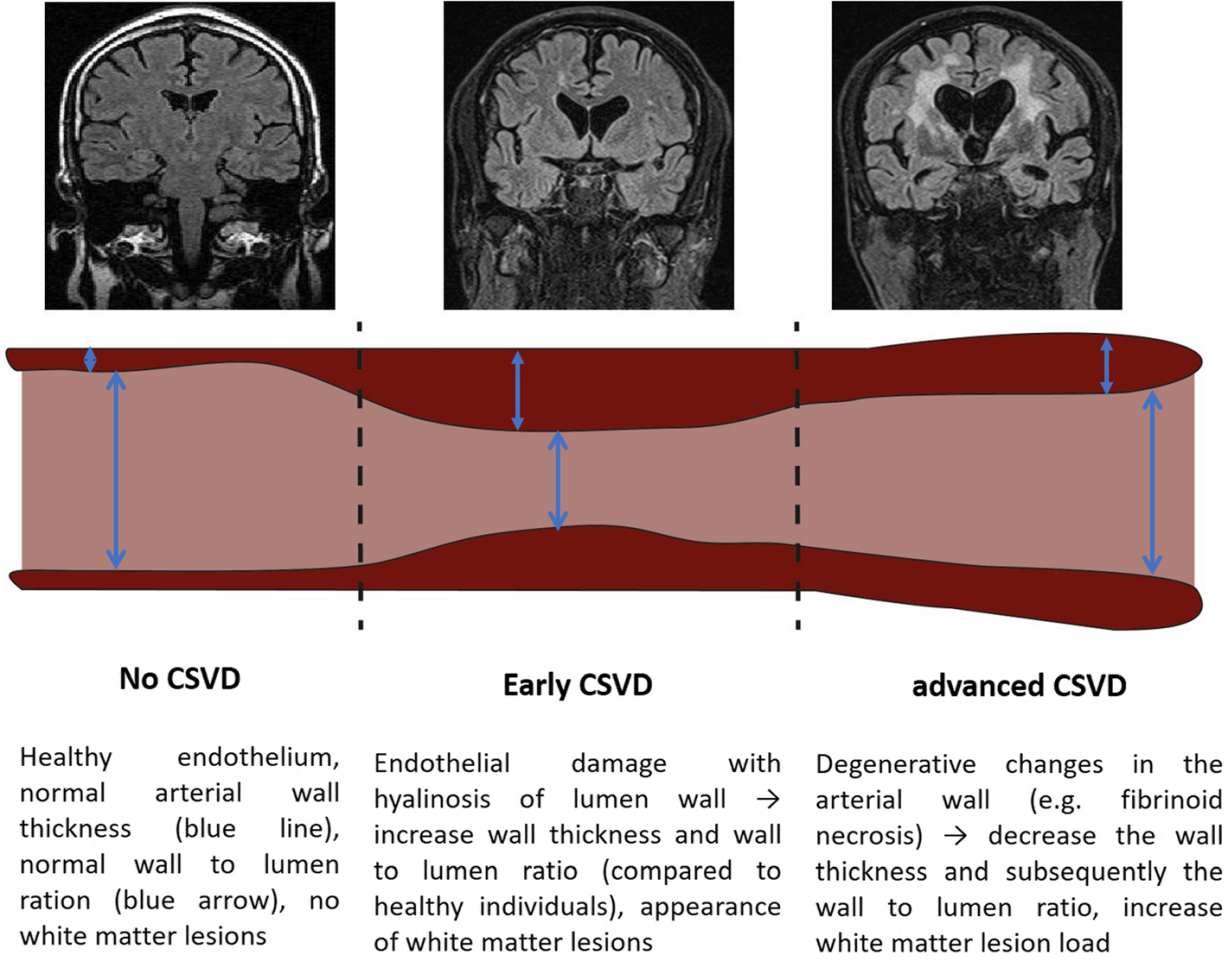
Model of retinal vessel changes over the course of cerebral small vessel disease (CSVD)

The characteristics of the retinal vessel were significantly different in a group of younger healthy individuals in the age-adjusted analyses. This observation is, however, not a proper comparison from a clinical point of view as age is a significant risk factor for CSVD. Figure 5 offers a model of retinal vessel changes along with the increase in the load of WMH that is proposed based on our findings. However, this model shall be interpreted with caution, as modifiable factors like antihypertensive treatment might influence it.

Our study suffers from several limitations, including the small sample size and lack of follow-up measurements of retinal vessels. Moreover, an age-matched group would be more suitable as a control group. Nevertheless, despite extensive efforts, it was not possible to gather a cohort of healthy individuals around 70 years old without cerebral white matter lesions, any vascular risk factors, and vascular or retinal diseases for our study. Thus, we included in this explorative pilot study younger healthy adults as a control group.

## Conclusions

In summary, our study might support the application of OCT-based retinal vessel assessment along with imaging and CSF-biomarker profiles to understand the pathophysiology and changes in cerebral small vessels in CSVD. The results of our explorative pilot study should be validated in independent prospective controlled studies.

## Supplementary information

**Additional file 1: Supplementary Figure 1**. The pathophysiology of sporadic cerebral small vessel disease (CSVD) includes vessel wall thickening, endothelial damage with disturbance of blood-brain barrier, microglial activation, astrogliosis and neuroaxonal demise (b). Magnetic resonance imaging (MRI) shows characteristic changes in the subcortical white matter, specifically extended white matter hyperintensities on fluid-attenuated inversion recovery (FLAIR) sequences (a). Combining the MRI, OCT-based assessment of the retinal arterioles (c), and a detailed CSF biomarker profile might help to reflect the CSVD pathology in vivo, which is the main aim of this exploratory work. **Supplementary Table 1**. Correlation between retinal vessel parameters, MRT findings, and CSF-biomarker parameters in *n* = 24 CSVD patients (Spearman’s r with *p*-value in brackets).

## Data Availability

The datasets generated during and/or analyzed during the current study are available from the corresponding author on reasonable request.

## Abbreviations

AD: Alzheimer’s Disease
CADASIL: Cerebral Autosomal Dominant Arteriopathy with Subcortical Infarcts and Leukoencephalopathy
CHI3L1: Chitinase-3-Like-1 protein
CSF: CerebroSpinal Fluid
CSVD: Cerebral Small Vessel Disease
CT: Computed Tomography
ECG: Electrocardiography
ELISA: Enzyme-linked Immunosorbent Assay
FLAIR: Fluid Attenuated Inversion Recovery
GFAP: Glial Fibrillary Acidic Protein
HCs: Young Healthy Controls
IWT: Inner Wall Thickness
LD: Lumen Diameter
LP: Lumbar Puncture
MMSE: Mini Mental State Examination
MRI: Magnetic Resonance Imaging
MWT: Mean Wall Thickness
OWT: Outer Wall Thickness
pNfH: Phosphorylated Neurofilament Heavy chain
SD-OCT: Spectral Domain Optical Coherence Tomography
SIMOA: Single Molecule Array
STRIVE: STandards for ReportIng Vascular changes on nEuroimaging
TIA: Transient Ischemic Attacks
WLR: Wall-to-Lumen Ratio
WMH: White Matter Hyperintensities
ZO-1: Zonula Occludens-1
