# Supplementary material for: Optical coherence tomography-based assessment of retinal vascular pathology in cerebral small vessel disease
Source: Neurol Res Pract. 2020 May 15;2:13. doi: 10.1186/s42466-020-00062-4 (PMC7650138; doi:10.1186/s42466-020-00062-4)
Supplement: Supplementary file 1 — Additional file 1: Supplementary Figure 1. The pathophysiology of sporadic cerebral small vessel disease (CSVD) includes vessel wall thickening, endothelial damage with disturbance of blood-brain barrier, microglial activation, astrogliosis and neuroaxonal demise (b). Magnetic resonance imaging (MRI) shows characteristic changes in the subcortical white matter, specifically extended white matter hyperintensities on fluid-attenuated inversion recovery (FLAIR) sequences (a). Combining the MRI, OCT-based assessment of the retinal arterioles (c), and a detailed CSF biomarker profile might help to reflect the CSVD pathology in vivo, which is the main aim of this exploratory work. Supplementary Table 1. Correlation between retinal vessel parameters, MRT findings, and CSF-biomarker parameters in n = 24 CSVD patients (Spearman’s r with p-value in brackets). [file 42466_2020_62_MOESM1_ESM.docx]

| 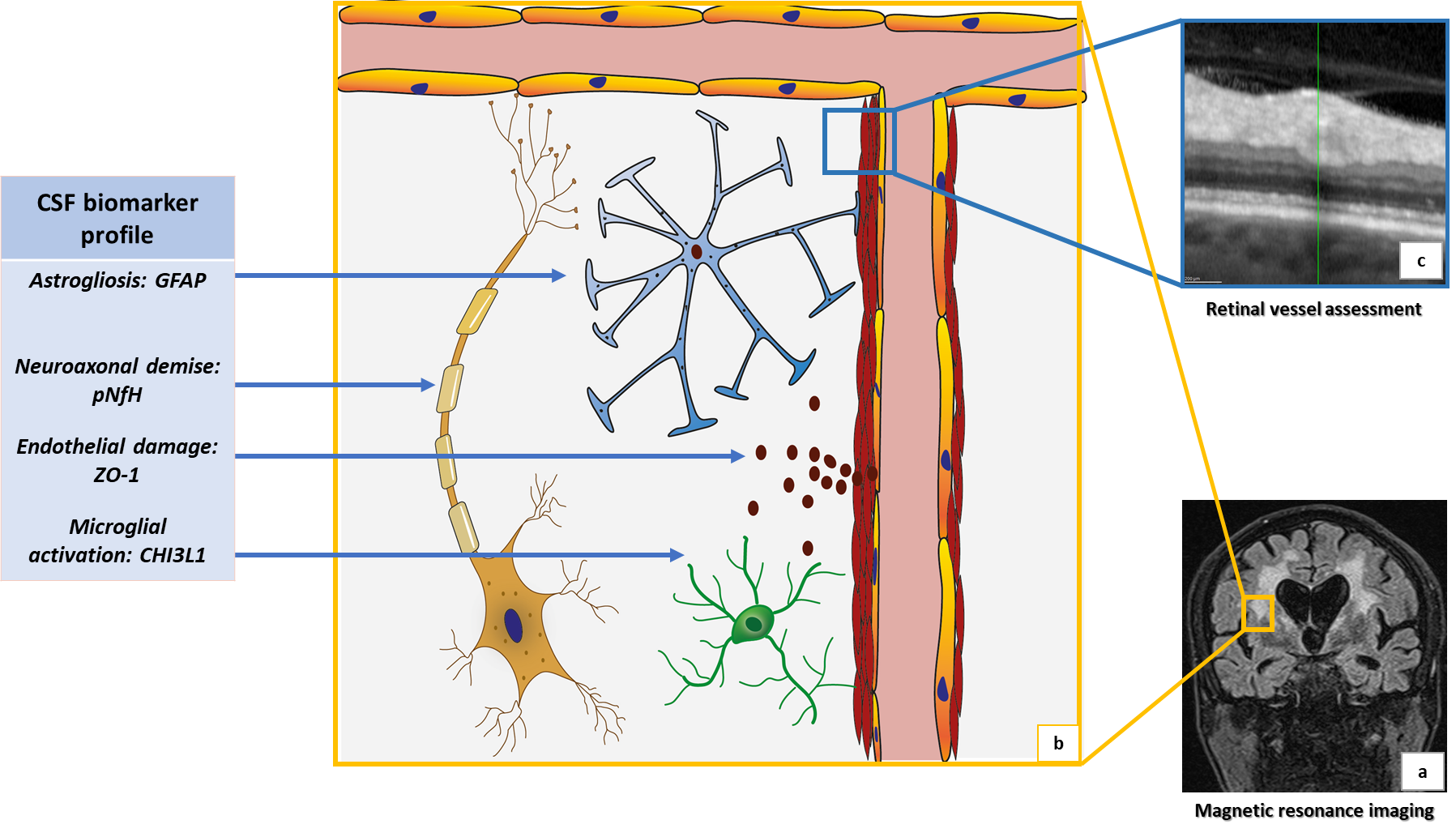 |
| --- |
| **Supplementary figure 1**: The pathophysiology of sporadic cerebral small vessel disease (CSVD) includes vessel wall thickening, endothelial damage with disturbance of blood-brain barrier, microglial activation, astrogliosis and neuroaxonal demise (b). Magnetic resonance imaging (MRI) shows characteristic changes in the subcortical white matter, specifically extended white matter hyperintensities on fluid-attenuated inversion recovery (FLAIR) sequences (a). Combining the MRI, OCT-based assessment of the retinal arterioles (c), and a detailed CSF biomarker profile might help to reflect the CSVD pathology in-vivo, which is the main aim of this exploratory work. |

| **Supplementary table 1: Correlation between retinal vessel parameters, MRT findings, and CSF-biomarker parameters in n=24 CSVD patients (Spearman's r with p-value in brackets).** | | | | | | | |
| --- | --- | --- | --- | --- | --- | --- | --- |
| **Retinal vessel parameters** | **White matter hyperintensities** | **Lacunar infarctions** | **Microbleeds** | **GFAP** | **pNfH** | **ZO-1** | **CHI3L1** |
| Mean arterial wall thickness (MWT) | - 0.4 (0.09) | 0.4 (0.09) | 0.4 (0.06) | **- 0.4 (0.03)** | - 0.3 (0.2) | - 0.3 (0.1) | - 0.4 (0.06) |
| Lumen diameter (LD) | 0.4 (0.09) | 0.2 (0.2) | -0.1(0.8) | 0.2 (0.5) | 0.3 (0.2) | 0.4 (0.07) | 0.4 (0.05) |
| Wall to lumen ration (WLR) | **- 0,5 (0.009)** | 0.1 (0.6) | 0.3 (0.2) | **- 0.4 (0.04)** | - 0.4 (0.06) | **- 0.5 (0.02)** | **- 0.6 (0.003)** |
| Total vessel diameter | **0.1 (0.001)** | 0.4 (0.07) | 0.1 (0.6) | -0.1 (0.6) | 0.04 (0.9) | 0.1 (0.5) | 0.1 (0.6) |
| GFAP: glial fibrillary acidic protein, pNfH: phosphorylated neurofilament heavy chain protein, ZO-1: zona occludens-1, CHI3L1: chitinase 3 like 1 | | | | | | | |
